# Alkaloids in Future Drug Discovery

**DOI:** 10.3390/molecules27041347

**Published:** 2022-02-16

**Authors:** Maria-José U. Ferreira

**Affiliations:** Research Institute for Medicines (iMed.ULisboa), Faculty of Pharmacy, Universidade de Lisboa, Av. Prof. Gama Pinto, 1649-003 Lisbon, Portugal; mjuferreira@ff.ulisboa.pt

Alkaloids are nitrogen-containing compounds, biosynthesized by both marine and terrestrial organisms, often with strong biological properties. They are among the largest classes of natural products and are found particularly in plants. The term alkaloid is related to their basic properties (alkali-like), due to the presence of one or more nitrogen atoms, as primary, secondary, or tertiary amines, frequently in heterocycles. Quaternary ammonium alkaloids are also found.

Characterized as bearing generally diverse and complex scaffolds, alkaloids are commonly classified based on the structure of the nitrogen-containing moiety. In alkaloid biosynthesis, the nitrogen atoms derive mostly from an amino acid precursor, whose carbon skeleton is generally maintained as a large extension in the alkaloid scaffold, giving rise to a different classification. However, in many alkaloids, sometimes named ‘pseudoalkaloids’, the nitrogen atom is incorporated into the structure, from non-amino acid precursors, at a late phase of the biogenetic process, through a transamination reaction [1].

This Special Issue entitled “Alkaloids in Future Drug Discovery” includes eleven articles, namely six original and five review articles, focused on biologically relevant natural alkaloids or semisynthetic derivatives.

Daley and Cordell [2] outlined the recognized biological impact of alkaloids on human health and the crucial role that they have long been playing in drug discovery. They highlighted the necessity of a new international paradigm to face the present challenges for treating human diseases globally. In order to obtain new therapeutic agents for facing diseases associated with multidrug resistance, in cancer cells and drug-resistant microorganisms, and to treat neglected diseases, the authors discussed the need for a new international strategy in natural product discovery and development, emphasizing the importance of extensive collaboration as the key to successful drug discovery programs.

Pandey et al. [3] summarized some features related to bisindole alkaloids isolated from *Alstonia*, a major genus of the Apocynaceae family that has been traditionally used to treat several diseases. They are able to biosynthesize a great variety of bioactive monoterpenoid indole alkaloids that can be found either as monomers or complex homo- or heterodimers, which are commonly more active than their corresponding monomeric units. Besides discussing bioactivity and biosynthesis features of bisindole alkaloids from *Alstonia* species, this review summarizes the synthesis of bisindole alkaloids bearing sarpagine, macroline, ajmaline, or pleiocarpamine units. The total synthesis of some indole alkaloids is also addressed.

Pruteanu et al. [4] reported a strategy for modifying diterpenes bearing the *ent*-kauranic scaffold. These transformations involved radical addition reactions at the *exo*-methylene moiety of *ent*-kaurenoic acid, including the azidoalkylation reaction. This reaction allows further functionalization of the diterpenic scaffold, increasing its structural complexity. With this strategy, the authors took advantage of the high structural diversity of diterpenes for generating synthetic diterpene-alkaloid hybrids having lactam and pyrrolidine pharmacophores. Some of them, containing *N*-heterocycles with a *spiro*-junction, have shown significant cytotoxicity against cancer cells with selective activity, exhibiting much higher IC_50_ values against normal non-cancerous cells.

There are several reports attributing antiviral activity to alkaloids. Fielding et al. [5] reviewed alkaloids with in vitro and in vivo antiviral activity against coronaviruses, with emphasis on human anti-coronaviral activity. According to this review, eleven alkaloids were reported in the literature with potential activity against severe acute respiratory syndrome coronavirus-2 (SARS-CoV-2), whose antiviral mechanisms are presented and discussed. Moreover, in silico analysis was also carried out by the authors aiming at studying the affinity of these alkaloids, with anti-coronavirus activity, for binding to the receptor-binding domain of SARS-CoV-2 spike protein, emphasizing the potential of these alkaloids against SARS-CoV-2.

According to Ayurveda, the medicinal plant *Holarrhena pubescens* (Apocynaceae), able to produce steroidal alkaloids, is one of the most common plants used for the treatment of diarrhea. Gupta et al. [6] evaluated the in vitro antibacterial activity of an alkaloid fraction of this plant against enterotoxigenic *Escherichia coli*, which showed significant antibacterial activity, thus validating its use in traditional medicine. Therefore, aiming at finding ligands that would interfere with the binding of the heat-stable enterotoxin secreted by enterotoxigenic *Escherichia coli* to the extracellular domain of guanylyl cyclase C, an intestinal receptor for heat-stable enterotoxins responsible for diarrhea, the authors carried out an in silico study with steroidal alkaloids from *Holarrhena pubescens*. They concluded that holadysenterine is a promising compound that deserves further in vitro and in vivo study.

Alzheimer’s disease is a progressive neurodegenerative disorder, and the most common type of dementia, which is characterized by a severe decrease in the function of the central cholinergic system. The incidence of Alzheimer’s disease, due the aging of the population, is increasing worldwide. The development of cholinesterase inhibitors is considered one of the most effective therapeutic approaches for Alzheimer’s disease treatment. Liu et al. [7] reported the chemical study of an extract of the scorpion Buthus *martensii* Karsch and the evaluation of the compounds isolated as acetylcholinesterase and butyrylcholinesterase inhibitors. The best results were obtained for buthutin A, a rare guanidine-type alkaloid that showed significant acetylcholinesterase inhibitory properties. The authors demonstrate that buthutin A was a mixed-type reversible inhibitor of acetylcholinesterase, with metal-binding properties, thus having potential health benefits.

Huperzine A is an alkaloid found in *Huperzia serrata* (Huperziaceae family), a medicinal plant that has long been used in Chinese traditional medicine to treat several ailments, including schizophrenia and Alzheimer’s disease. Huperzine A, which has shown memory-enhancing effects, was approved for clinical use in China and is currently used as a supplement for avoiding memory deterioration in the USA. These memory benefits have been attributed to its acetylcholinesterase inhibitory properties. Therefore, there has been an increasing demand for huperzine A that cannot be provided by *Huperzia serrata* due to its low content in this compound, leading researchers to seek alternative resources. According to plant–microbe interaction studies, endophytic fungi play important roles in plant growth. Cui et al. [8] studied the diversity of the endophytic fungal community within *H. serrata* and its role to the production of huperzine A by the host plant.

Plants from the Amaryllidaceae family have been used traditionally for nervous system-related disorders. They are well-known for biosynthesising bioactive Amaryllidaceae-type alkaloids, which have shown several biological activities including neuroprotective properties. Galanthamine is an Amaryllidaceae alkaloid approved by the Food and Drug Administration for the treatment of Alzheimer’s disease. Omoruyi et al. [9] reported the phytochemical study of the bulbs of *Crossyne flava* (Amaryllidaceae family) and investigated the neuroprotective activities of its extract and isolated alkaloids, namely pancratinine B, bufanidrine, buphanisine, and epibuphanisine, in an in vitro model of Parkinson’s disease. The results obtained in this study were in agreement with the use of plants of Amaryllidaceae family in traditional medicine for treating nervous system-related disorders.

Metabolic syndrome constitutes a group of diverse risk factors that together increase the possibility of developing cardiovascular diseases and diabetes mellitus type 2. Aporphines are one of the major groups of isoquinoline alkaloids that were reported to have protective effects on the different risk factors that characterize the metabolic syndrome. Wang et al. [10] reviewed the role of aporphine alkaloids in the prevention and treatment of metabolic syndrome, whose incidence and prevalence has increased worldwide. The potential mechanisms of action of aporphine alkaloids, in metabolic syndrome, were also addressed.

Pyrrolizidine alkaloids, characterized as bearing a bicyclic skeleton and generally occurring as esters, are known to demonstrate marked hepatic toxicity in humans and animals. Their toxicity is related with the presence of a double bond in the pyrrolizidine scaffold. Unsaturated pyrrolizidine alkaloids are transformed in the liver into pyrrolic metabolites, which are strong alkylating agents able to react with nucleophiles, making them more toxic when compared to saturated pyrrolizidines. However, in spite of their toxicity, several plants containing pyrrolizidine alkaloids have been used in traditional medicine in several countries. Some of them have shown anti-microbial, anti-inflammatory, anti-cancer and anti-viral properties. Wei et al. [11] reviewed the biological properties of these compounds.

Plants bearing benzophenanthridine alkaloids have long been used in traditional medicine for treating different disorders. Benzophenanthridines are a group of tetracyclic plant-derived secondary metabolites, belonging to the benzylisoquinoline class of alkaloids, which are mostly found mostly plants from the Fumariaceae, Papaveraceae and Rutaceae families. They are characterized by a broad range of biological properties that have attracted increasing attention owing to their potential pharmacological use. Laines-Hidalgo et al. [12] reviewed the new advances in benzophenanthridines biosynthesis and physiological roles. Their industrial applications, mainly with pharmacological approaches, were also addressed.

Owing to their huge chemical diversity and complexity, coupled with their broad range of biological activities, alkaloids have long deserved great interest from natural product chemists and medicinal chemists. Both the original and review works included in this Special Issue clearly highlight the important role in drug discovery of this group of natural products, bearing privileged scaffolds with promising benefits to human health.

## References

[1] Dewick P.M., Dewick P.M. (2009). Medicinal Natural Products: A Biosynthetic Approach.

[2] Daley S.K., Cordell G.A. (2021). Alkaloids in contemporary drug discovery to meet global disease needs. Molecules.

[3] Pandey K.P., Rahman M.T., Cook J.M. (2021). Bisindole alkaloids from the alstonia species: Recent isolation, bioactivity, biosynthesis, and synthesis. Molecules.

[4] Pruteanu E., Gîrbu V., Ungur N., Persoons L., Daelemans D., Renaud P., Kulcițki V. (2021). Preparation of antiproliferative terpene-alkaloid hybrids by free radical-mediated modification of ent-kauranic derivatives. Molecules.

[5] Fielding B.C., da Silva Maia Bezerra Filho C., Ismail N.S.M., de Sousa D.P. (2020). Alkaloids: Therapeutic potential against human coronaviruses. Molecules.

[6] Gupta N., Choudhary S.K., Bhagat N., Karthikeyan M., Chaturvedi A. (2021). In silico prediction, molecular docking and dynamics studies of steroidal alkaloids of holarrhena pubescens wall. ex G. don to guanylyl cyclase C: Implications in designing of novel antidiarrheal therapeutic strategies. Molecules.

[7] Liu Y.M., Fan J.J., Wang L.N. (2021). Discovery of guanidine derivatives from buthus martensii karsch with metal-binding and cholinesterase inhibition properties. Molecules.

[8] Cui L., Noushahi H.A., Zhang Y., Liu J., Cosoveanu A., Liu Y., Yan L., Zhang J., Shu S. (2021). Endophytic fungal community of huperzia serrata: Diversity and relevance to the production of huperzine a by the plant host. Molecules.

[9] Omoruyi S.I., Ibrakaw A.S., Ekpo O.E., Boatwright J.S., Cupido C.N., Hussein A.A. (2021). Neuroprotective activities of crossyne flava bulbs and amaryllidaceae alkaloids: Implications for parkinson’s disease. Molecules.

[10] Wang F.X., Zhu N., Zhou F., Lin D.X. (2021). Natural aporphine alkaloids with potential to impact metabolic syndrome. Molecules.

[11] Wei X., Ruan W., Vrieling K. (2021). Current knowledge and perspectives of pyrrolizidine alkaloids in pharmacological applications: A mini-review. Molecules.

[12] Laines-Hidalgo J.I., Muñoz-sá J.A., Loza-müller L., Vá F. (2022). An update of the sanguinarine and benzophenanthridine alka- loids biosynthesis and their applications. Molecules.

